# A novel virus genome discovered in an extreme environment suggests recombination between unrelated groups of RNA and DNA viruses

**DOI:** 10.1186/1745-6150-7-13

**Published:** 2012-06-11

**Authors:** Geoffrey S Diemer, Kenneth M Stedman

**Affiliations:** 1 Department of Biology, and the Center for Life in Extreme Environments, Portland State University, 1719 SW 10th Avenue, SRTC room 246, Portland, OR, 97201, USA

**Keywords:** Non-retroviral RNA virus integration, RNA-DNA recombination, Viral metagenomics, Metaviromics, Virus ecology, Viral diversity, Modular theory of virus evolution, Interviral lateral gene transfer, RNA World, DNA World, Virus World

## Abstract

**Background:**

Viruses are known to be the most abundant organisms on earth, yet little is known about their collective origin and evolutionary history. With exceptionally high rates of genetic mutation and mosaicism, it is not currently possible to resolve deep evolutionary histories of the known major virus groups. Metagenomics offers a potential means of establishing a more comprehensive view of viral evolution as vast amounts of new sequence data becomes available for comparative analysis.

**Results:**

Bioinformatic analysis of viral metagenomic sequences derived from a hot, acidic lake revealed a circular, putatively single-stranded DNA virus encoding a major capsid protein similar to those found only in single-stranded RNA viruses. The presence and circular configuration of the complete virus genome was confirmed by inverse PCR amplification from native DNA extracted from lake sediment. The virus genome appears to be the result of a RNA-DNA recombination event between two ostensibly unrelated virus groups. Environmental sequence databases were examined for homologous genes arranged in similar configurations and three similar putative virus genomes from marine environments were identified. This result indicates the existence of a widespread but previously undetected group of viruses.

**Conclusions:**

This unique viral genome carries implications for theories of virus emergence and evolution, as no mechanism for interviral RNA-DNA recombination has yet been identified, and only scant evidence exists that genetic exchange occurs between such distinct virus lineages.

**Reviewers:**

This article was reviewed by EK, MK (nominated by PF) and AM. For the full reviews, please go to the Reviewers' comments section.

## Background

While viruses are known to be the most abundant organisms on earth, their collective evolutionary history, biodiversity and functional capacity is poorly understood [1-3]. Despite many inherent obstacles, viral metagenomics is enabling a more detailed evaluation of environmental viral diversity, and is burgeoning as an important tool for studying virus evolution [4-7]. Perhaps the greatest impediment to the evolutionary study of viruses is that there is no single phylogenetic marker in common amongst all viruses. Instead, “virus hallmark genes” [8] that are present in sub-groups of viruses are often used as the basis for taxonomic classification and evolutionary studies. However, lateral gene transfer between viruses complicates these analyses and virus classification schemes are likewise intensely debated [9-12].

The known virosphere consists of three principal viral types; the RNA-only viruses, which do not require a DNA intermediate in the replication cycle, viruses with DNA-based genomes, and retroid viruses that require the reverse transcription of their RNA into DNA during the virus life-cycle [12]. Lateral exchange of viral genes, via multiple possible mechanisms, is rampant among viruses within each of these principal types, but is generally confined to closely related viruses, or viruses (and plasmids) with similar replication mechanisms [13-20]. Clear examples of recent lateral gene transfer (LGT) from RNA-only to DNA-only viral types have not been observed.

We report the discovery of a group of circovirus-like DNA genomes whose common ancestor appears to have incorporated a capsid protein (CP) gene known previously only in RNA viruses. The mechanism responsible for the integration of the RNA virus cistron into the DNA virus, and the point in evolutionary time at which it occurred, are unclear. Relatively low levels of sequence divergence between the homologous viral proteins indicate that the recombination event took place relatively recently. Moreover, it suggests that entirely new virus types may emerge via the lateral exchange of functional and structural modules from viruses of vastly different types, utilizing as yet unknown mechanisms.

## Results and discussion

### Analysis and overview

A metagenomics approach was used to investigate virus diversity in Boiling Springs Lake (BSL) located in Lassen Volcanic National Park, USA. BSL is an acidic, high temperature lake (ranging between 52°C and 95°C, with a pH of approximately 2.5), which sustains a purely microbial ecosystem comprised of novel *Archaea, Bacteria* and several species of unicellular *Eukarya*[21,22].

Initial analysis of the individual metagenomic DNA sequences from virus-sized particles obtained from BSL indicated the presence of a virus capsid protein (CP) gene related to the downy mildew-infecting *Sclerophthora macrospora-*A (SmV-A) and *Plasmopara halstedii-*A (PhV-A) viruses [23,24]. SmV-A and PhV-A are unclassified linear, multipartite ssRNA viruses encoding capsid proteins similar to the plant-infecting *Tombusviridae*[24]. The BSL metagenomic sequences were then assembled into contigs and, surprisingly, a putative rolling circle replicase protein (Rep) gene most closely related to the circular ssDNA *Circoviridae* Rep was located immediately upstream of the ssRNA virus-like CP gene.

The complete circular genome was subsequently amplified from a native BSL DNA sample by inverse PCR using primers within the CP open reading frame (ORF). A native BSL DNA template was chosen for inverse PCR that was not used for metagenomic sequencing and was not pre-amplified with ϕ29 polymerase, in order to rule out the possibility of spurious chimerism during sample preparation or sequence assembly. Sanger sequencing of the cloned viral genome was performed to confirm the original metagenomic sequence, to verify circularity, and to allow ORF prediction.

Translations of the predicted open reading frames for CP and Rep were used as search query sequences to compile a set of related virus genes from publicly available sequence databases. Individual phylogenies of the putative CP and Rep proteins were established using a subset of the search output sequences. Putative tertiary protein structures for Rep and CP were predicted by threading the ORF translations to homologous proteins with solved crystallographic structures.

## Results

**Figure 1:**
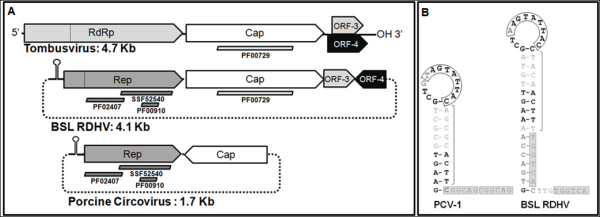
**Organiztion of the BSL RDHV genome.(A)***Schematic representations of tombusvirus, BSL RDHV and porcine circovirus (PCV) genomes:* Tombusviruses are linear ssRNA viruses, BSL RDHV and PCV are circular ssDNA viruses. Bars below ORFs indicate protein families detected by InterProScan.** (B) ***Comparison of PCV-1 and BSL RDHV stem-loops:* Both BSL RDHV and circoviruses have a conserved stem-loop in the intergenic region upstream of Rep. Black text indicates sequence identity. Nonanucleotide replication origins in the 13nt loop are boxed. Brackets indicate the 9 nucleotide stem preceding hexamer repeats H1 and H2 (gray boxes).

Since the novel BSL virus genome harbors genes homologous to both ssRNA and ssDNA viruses, it will be provisionally referred to herein as an “RNA-DNA hybrid virus”, abbreviated “RDHV”. Although the BSL RDHV genome is circular, the size of the genome is roughly double that of typical circoviruses, and the ORFs are arranged in an uncommon orientation (Figure 1A). The BSL RDHV Rep contains an N-terminal rolling circle replicase endonuclease (RCRE) domain (PF02407) [25] and a C-terminal superfamily-3 RNA helicase (S3H) domain (PF00910), both of which are found in circoviral Reps [26]. A highly conserved DNA stem-loop in the intergenic region upstream of Rep is found in both BSL RDHV and porcine circoviruses [27] (Figure 1B). The CP gene, however, is similar to those of the small icosahedral monopartite (+)ssRNA tombusviruses (PF00729).

**Figure 2:**
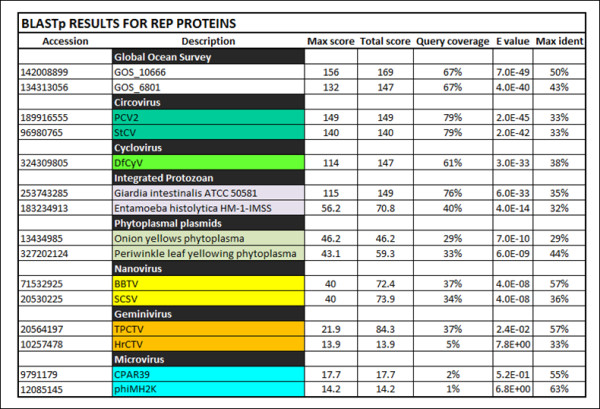
**BLASTp data for Rep amino acid sequences.** The BSL RDHV Rep ORF amino acid sequence was compared to related Rep sequences using BLASTp. Output parameters are shown. Virus family designations are indicated when possible. (GOS) Global Ocean Survey paired-end read scaffolds 10666 and 6801. (PCV2) Porcine Circovirus-2, (StCV) Starling Circovirus, (DfCyV) Dragonfly Cyclovirus. (BBTV) Banana Bunchy Top Virus, (SCSV) Subterranean Clover Stunt Virus. (TPCTV) Tomato Pseudo Curly Top Virus, (HrCTV) Horseradish Curly Top Virus.

**Figure 3:**
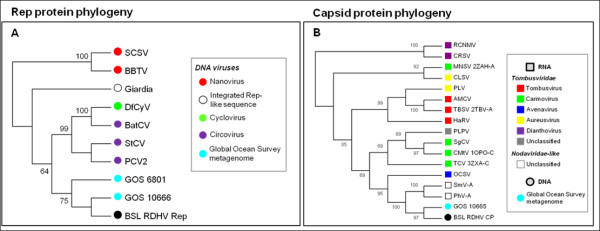
**Rolling circle replicase (Rep) and capsid protein (CP) phylogenies.(A)** The Rep protein phylogeny was inferred from 204 conserved amino acid positions using the Neighbor-Joining method. Bootstrap values above a 60% significance threshold, based on 1000 replicates, are shown next to the branches. *NANOVIRIDAE:* (SCSV) Subterranean Clover Stunt Virus, (BBTV) Banana Bunchy Top Virus. *CIRCOVIRIDAE:* (DfCyV) Dragonfly Cyclovirus, (BatCV) Bat Circovirus, (StCV) Starling Circovirus, (PCV2) Porcine Circovirus-2. (GOS) Global Ocean Survey paired-end read scaffolds 6801 and 10666. **(B)** The capsid protein (CP) phylogeny was inferred from 256 conserved amino acid positions using the Neighbor-Joining method. Bootstrap values above a 60% significance threshold, based on 1000 replicates, are shown next to the branches. *TOMBUSVIRIDAE: Dianthoviruses;* (RCNMV) Red Clover Necrotic Mosaic Virus, (CRSV) Carnation Ringspot Virus. *Aureusviruses:* (CLSV) Cucumber Leaf Spot Virus, (PLV) Pothos Latent Virus. *Tombusviruses;* (AMCV) Artichoke Mottled Crinkle Virus, (TBSV) Tomato Bushy Stunt Virus, (HaRV) Havel River Tombusvirus. *Unclassified Tombusviridae;* (PLPV) Pelargonium Line Pattern Virus. *Carmoviruses;* (MNSV) Melon Necrotic Spot Virus, (SgCV) Saguaro Cactus Virus, (CMtV) Carnation Mottle Virus, (TCV) Turnip Crinkle Virus. *Avenavirus;* (OCSV) Oat Chlorotic Stunt Virus. *NODAVIRIDAE-like:* (SmV-A) *Sclerophthora macrospora* Virus-A, (PhV-A) *Plasmopara halstedii* Virus-A. (GOS) Global Ocean Survey paired-end read sequence 10665. Virus proteins for which structures are available are listed with the Protein Data Bank (PDB) identification code after the virus abbreviation.

**Figure 4:**
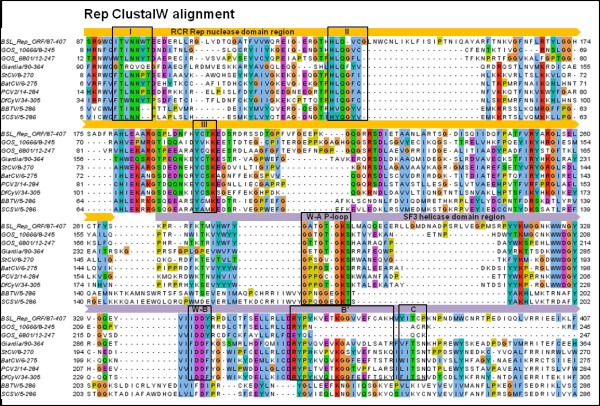
**Rolling circle replicase (Rep) amino acid multiple sequence alignment.** The BSL RDHV Rep ORF sequence (BSL_Rep_ORF) is aligned to closely related sequences retrieved from NCBI using PSI-BLAST searches. Sequence alignment was performed using ClustalW set to default parameters in MEGA v.5. The endonuclease domain motifs I, II and III, and superfamily-3 helicase domain Walker A P-loop NTPase (W-A P-loop), Walker B (W-B), B’ and C motifs are boxed and labeled. Rep nuclease (RCRE) and superfamily-3 helicase (S3H helicase) domains are indicated by block arrows above the sequence alignment. A ClustalW color scheme is applied to the multiple alignment using Jalview. The range of amino acid residues used in the alignment are shown after the sequence name. (GOS) Global Ocean Survey paired-end read scaffolds 10666 and 6801. *CIRCOVIRIDAE:* (PCV2) Porcine Circovirus-2, (StCV) Starling Circovirus, (BatCV) Bat Circovirus, (DfCyV) Dragonfly Cyclovirus. *NANOVIRIDAE:* (BBTV) Banana Bunchy Top Virus, (SCSV) Subterranean Clover Stunt Virus.

BLAST searches and phylogenetic analysis indicate that the BSL RDHV Rep is more closely related to circoviral Reps than to those of other viruses or plasmids (Figures 2 and 3A). Amino acid sequence alignments indicate significant conservation in both RCRE and S3H Rep domains (Figure 4). The N-terminal RCRE domain contains well-conserved motifs I, II and III. The putative α3-helix contains the motif III (YxxK) active-site tyrosine [28-31]. The S3H domain also contains well-conserved Walker-A, Walker-B, B’ and C motifs [32-35]. These analyses, combined with the circularity of the genome and the presence of a circovirus-like DNA stem loop preceding Rep, indicate that the BSL isolate is a circovirus-like entity [26] and imply that the packaged genome is comprised of ssDNA.

**Figure 5:**
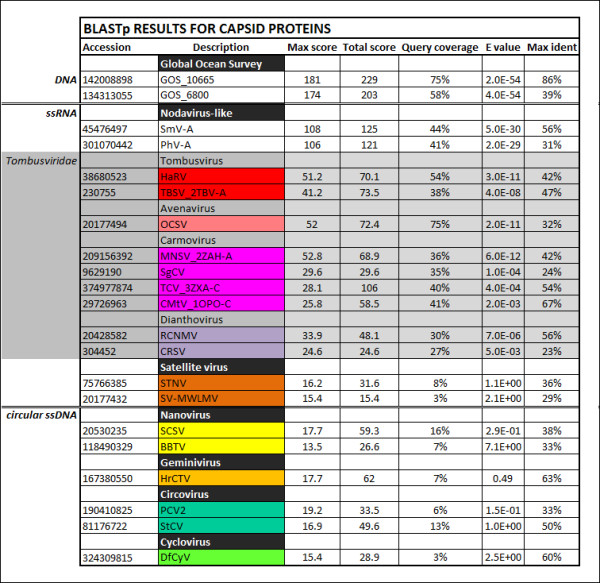
**BLASTp data for capsid protein (CP) amino acid sequences.** The BSL RDHV CP ORF amino acid sequence was compared to related CP sequences using BLASTp. Output parameters are shown. Virus family designations are indicated when possible. (GOS) Global Ocean Survey paired-end read sequences 10665 and 6800. (SmV-A) *Sclerophthora macrospora* Virus-A, (PhV-A) *Plasmopara halstedii* Virus-A. (HaRV) Havel River Tombusvirus, (TBSV) Tomato Bushy Stunt Virus, (OCSV) Oat Chlorotic Stunt Virus, (MNSV) Melon Necrotic Spot Virus, (SgCV) Saguaro Cactus Virus, (TCV) Turnip Crinkle Virus, (CMtV) Carnation Mottle Virus, (RCNMV) Red Clover Necrotic Mosaic Virus, (CRSV) Carnation Ringspot Virus, (STNV) Satellite of Tobacco Necrosis Virus, (SV-MWLMV) Satellite Virus of Maize White Line Mosaic Virus, (SCSV) Subterranean Clover Stunt Virus, (BBTV) Banana Bunchy Top Virus, (HrCTV) Horseradish Curly Top Virus, (PCV2) Porcine Circovirus-2, (StCV) Starling Circovirus, (DfCyV) Dragonfly Cyclovirus.

BLAST searches and phylogenetic analysis indicate that the BSL RDHV capsid protein groups with the CPs of ssRNA viruses with multipartite genomes (SmV-A and PhV-A) and the monopartite ssRNA *Tombusviridae,* to the exclusion of capsid proteins found in ssDNA circoviruses, and plant-infecting nanoviruses and geminiviruses that also encode Rep (Figures 3B and 5). The SmV-A and PhV-A viruses are taxonomically categorized as unclassified Noda-like viruses. This assessment is based primarily on the RNA-dependent RNA-polymerase (RdRp) sequence and not the CP [36], which was apparently acquired from a tombusvirus-like ancestor [24]. As Melon Necrotic Spot Virus and several other tombusviruses are transported to the plants they infect by fungal vectors, it is likely that the multipartite SmV-A and PhV-A, which infect downy mildews of plants, incorporated an RNA transcript encoding a tombusvirus-like CP.

**Figure 6:**
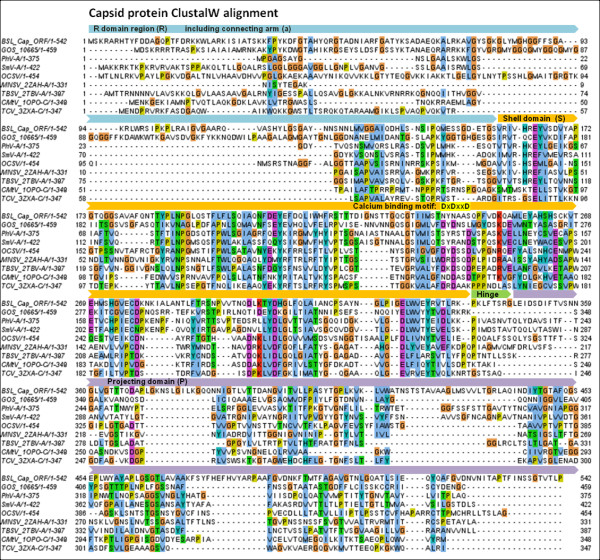
**Capsid protein (CP) amino acid multiple sequence alignment.** The BSL RDHV CP ORF sequence (BSL_Cap_ORF) is aligned to closely related sequences retrieved from PSI-BLAST searches using ClustalW set to default parameters in MEGA v.5. The R, a, S, h and P domain regions are indicated by block arrows above the sequence alignment. A ClustalW color scheme is applied to the multiple alignment using Jalview. (GOS) Global Ocean Survey paired-end read scaffold 10665. *NODAVIRIDAE-like:* (PhV-A) *Plasmopara halstedii* Virus-A, (SmV-A) *Sclerophthora macrospora* Virus-A. *TOMBUSVIRIDAE: Avenavirus;* (OCSV) Oat Chlorotic Stunt Virus. *Carmoviruses;* (MNSV) Melon Necrotic Spot Virus, (CMtV) Carnation Mottle Virus, (TCV) Turnip Crinkle Virus. *Tombusvirus;* (TBSV) Tomato Bushy Stunt Virus. The range of amino acid residues used in the alignment are shown after the sequence name. Virus proteins for which structures are available are listed with the Protein Data Bank (PDB) identification code after the virus abbreviation.

Several *Tombusviridae* capsid protein structures have been solved, including the ssRNA Tomato Bushy Stunt (TBSV) and Melon Necrotic Spot (MNSV) tombusviruses [37,38]. These structures have characteristic shell (S) and projecting (P) domains that are linked by a short hinge [39]. The N-terminal region that precedes the S-domain is referred to as the RNA-interacting, or R domain. The R domain, which allows the CP to interact with the viral RNA in the interior of the virion, is generally found to be unstructured and to contain basic, nucleic acid-interacting residues [37]. The connecting arm region (a), between the R and S domains, forms a β-annulus structure that connects three CPs at the 3-fold axes of symmetry in the virion [40]. A ClustalW multiple sequence alignment of the BSL and related CPs demonstrates different levels of conservation in each of the CP domain regions (Figure 6). The greatest level of sequence conservation is found within the S domain of these proteins [41]. Many of the *Tombusviridae* also contain a calcium ion binding motif (DxDxxD) in the S-domain interaction region [42,43] that is thought to aid in uncoating of the virus when it enters the low calcium environment of the plant cell cytoplasm [44]. Replacing aspartic acids D155 and D157 with asparagines (N) in the motif created a calcium ion binding-deficient mutant of the Turnip Crinkle Virus (TCV) CP [44]. Remarkably, the very same amino acid substitutions are present in the BSL RDHV capsid protein (Figure 6).

**Figure 7:**
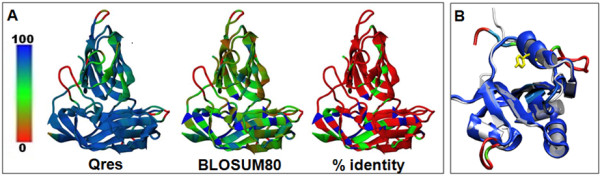
**BSL RDHV predicted protein structures.(A)***Structural analysis of the BSL RDHV capsid protein*: The predicted structure for residues 156–302 of the BSL RDHV capsid protein monomer ORF is overlaid on the Tomato Bushy Stunt Virus (PDB ID: 2TBV) and Melon Necrotic Spot Virus (PDB ID: 2ZAH) capsid proteins (latter two are not shown). The predicted BSL RDHV capsid is color-coded by the “Qres” dimensional structure-fitting parameter score, by BLOSUM80, and by percent amino acid sequence identity. **(B)***Structural analysis of the BSL RDHV replicase catalytic core*: The predicted BSL RDHV Rep nuclease domain structure (colored by Qres) threaded onto PCV2 Rep (PDB ID: 2HW0) in silver. The conserved active-site tyrosine is shown in yellow.

Structural similarity was assessed for the CP and Rep proteins by threading the BSL RDHV sequences to homologous solved structures. To substantiate the hypothesis that the BSL RDHV CP conforms to the S-P domain configuration, the BSL RDHV CP structure was predicted by threading using the known structures of the MNSV (PDB ID: 2ZAH) and TBSV (PDB ID: 2TBV) CPs (Figure 7A). The Z-scores for the structure predictions were 71.1 and 54.4, respectively (a Z-score above 10 indicates a high reliability model). The predicted structure of the BSL RDHV capsid protein was highly congruent to the S-P domain architecture found in the ssRNA TBSV and MNSV tombusviruses. The S domain is a canonical β-barrel jelly-roll fold consisting of nine antiparallel β-strands and two α-helices located between the β-2 / β-3, and β-4 / β-5 strands [45]. The P-domain is in a β-barrel configuration composed of 8 antiparallel β -strands, with an additional β-turn between the hinge and P-domain. No α-helices are present in the P-domain. The BSL RDHV is the only known DNA virus harboring the putative S-P domain architecture, which is otherwise found exclusively in ssRNA viruses.

Structures for the BSL Rep nuclease and S3H regions were also predicted with high confidence based on the circular ssDNA Porcine Circovirus-2 (PCV2) Rep nuclease domain (PDB ID: 2HW0, Z-score = 128) [30] (Figure 7B) and the papillomavirus E1 multimeric helicase domain (PDB ID: 1TUE, Z-score = 68) [46]. The predicted nuclease domain of the BSL RDHV Rep contains the active-site YxxK motif in the α3 helix, similar to other viral Reps [47,48] (Figure 7B). The predicted structure of the BSL RDHV Rep helicase domain is capable of being appropriately assembled *in silico* into a complete hexameric unit (data not shown).

Due to a lack of detectable sequence similarity, functions for BSL RDHV ORF-3 or ORF-4 cannot be proposed. ORF-3 and ORF-4 are not homologues of the tombusvirus ORFs with the same designation, as depicted in Figure 1A.

### Identifying similar viruses in other environments

To determine whether the BSL RDHV is endemic to Boiling Springs Lake, or whether it represents a larger group of viruses, environmental sequence databases were scanned for homologous CP and Rep sequences arranged in similar configurations. Sequences of both proteins were found to be similar to translated metagenomic DNA sequences derived from the Global Ocean Survey (GOS) [49]. The BSL RDHV Rep protein sequence was also similar to *Entamoeba* and *Giardia* integrated Rep-like sequences, possibly acquired from viruses or plasmids [50] (Figures 23A and 4).

Three candidate BSL RDHV-like genomes were detected in marine environments. Although many GOS sequences are similar to either the BSL RDHV CP or Rep proteins, only two paired-end read scaffolds from the GOS contain both circovirus-like Rep and tombusvirus-like CP genes similar to the BSL RDHV (GOS 10665–10666 and GOS 6800–6801; GI:142008897 and GI:134313054, respectively) (Figures 2 through 6). The GOS 6800 CP sequence is truncated and was thus not used in the multiple alignment or phylogenetic analysis, however the available sequence was sufficient for BLASTp comparison (Figures 3B5 and 6). The GOS 6801 Rep sequence (GI:142008897 / EBA57255.1), while similar to the BSL RDHV, also contains a putative parvoviral NS1 protein fold that has been identified in a number of marine metagenome circoviral Rep sequences [51], possibly indicating a history of lateral gene exchange between linear (parvoviral) and circular ssDNA virus groups. A shotgun sequence from the Sargasso Sea [52] (GI:129569619) was also identified, which contains contiguous N and C-terminal fragments of Rep and CP that are similar to the BSL RDHV sequences and are in the same orientation (data not shown). These data strongly suggest that previously undetected BSL RDHV-like viruses are widespread in the marine environment, and are likely to be found in other environments as well.

## Conclusions

A parsimonious scenario explaining the provenance of the BSL RDHV and its relatives is that a DNA circovirus-like progenitor acquired a capsid protein gene from a ssRNA virus via reverse transcription and recombination. While the mechanisms responsible for interviral RNA-RNA and DNA-DNA recombination are well characterized [53-57], no mechanism has yet been proposed to account for the inferred instances of interviral RNA-DNA recombination [58-60]. Any instance in which an RNA cistron is converted into DNA and then integrated into a DNA genome presumably involves a reverse-transcriptase (RT) mechanism. However, no trace of a RT module exists in the BSL RDHV. The presence of non-retroviral RNA virus genes in cellular genomes [61-66] suggests that some cellular mechanism exists that allows RNA-DNA recombination in lieu of a virus-derived RT. Although the group II intron retro-homing phenomenon [67] and transposon mediated exchanges have not been observed to mediate interviral lateral gene transfer, these or similar host cell-based mechanisms may have facilitated the formation of the BSL RDHV-like viruses. Moreover, the ancestral host must have also been permissive to both circovirus-like DNA viruses and plant (or fungal)-like ssRNA viruses for interviral RNA-DNA recombination to have occurred.

As more viral metagenomic data are generated and analyzed, additional evidence of recombination between RNA and DNA virus groups will likely be discovered. Such findings would highlight the intriguing possibility that novel virus groups can emerge via recombination between highly disparate virus types. However, a dearth of similar examples of interviral RNA-DNA recombination would otherwise suggest that such events are either rare or perhaps ancient.

Considering the possibility that the recombination mechanism by which DNA viruses acquire RNA cistrons is ancient, it would have broad implications for the early evolution of viruses. As RNA viruses are believed to evolutionarily precede the emergence of DNA viruses [8,68], determining the mechanism responsible for direct recombination between RNA and DNA viruses may help address how genes from the “RNA World” were first incorporated into nascent DNA-based genomes during the putative “Virus World” era, and thus further implicate viruses in the RNA-World to DNA-World transition [68-71]. In any case, the discovery of the BSL RDHV-like virus group extends the modular theory of virus evolution [8,72-74] to encompass a much broader range of possibilities than previously thought.

## Methods

### Metagenomic sample preparation

Briefly, pore water from 20 liters of BSL sediment was concentrated to 30 mL by tangential flow filtration (TFF) at a molecular weight cut-off of 100 kDa. The native pore water in the concentrate was then exchanged with SM phage buffer (100 mM NaCl, 10 mM MgCl_2_ and 50 mM Tris-Base, adjusted to pH 7.0) using TFF to a final volume of 30 mL. The virus concentrate was split into two 15 mL aliquots. One of the 15 mL concentrates was centrifuged for 30 min. at 3,000 x g in an attempt to remove microorganisms and spores. The supernatant was then DNase-treated to remove extraneous DNA. Virus-sized particles in solution were disrupted by treatment in 10% SDS and 20 mg/mL Proteinase K. A 1:15 volume of CTAB (hexadecyltrimethyl ammonium bromide)/NaCl solution (0.125 g/mL CTAB in 2 M NaCl) was added to the digest to reduce dissolved cellulosic material. Following phenol-chloroform-isoamyl alcohol (25:24:1) extraction, DNA was precipitated from the aqueous layer using 0.6 volumes of −20°C isopropanol. The resulting DNA was tested for microbial DNA contamination using the 515 F/1492R universal 16S rRNA gene primer set [75]. Microbial contamination of the sample was determined to be too high for use in metagenomic sequencing (data not shown). The remaining 15 mL of BSL virus concentrate was filtered using a Minisart 200 nm pore SFCA syringe filter (Sartorius-Stedim Biotech), prior to DNA extraction using the method described above. DNA extracted from virus-sized particles (≪ 200 nm) was amplified with ϕ29 polymerase [76] (GenomiPhi v2, GE Healthcare Life Sciences). The resulting DNA was tested for microbial DNA contamination using 16S rRNA gene PCR, as described above, and was found to be virtually free of microbial DNA. This BSL DNA sample was sequenced at the Broad Institute using Roche 454 FLX Titanium reagents as part of the Gordon and Betty Moore Foundation’s Marine Microbiology Initiative [77].

### Bioinformatic analysis

Analysis by tBLASTx [78] of the *ca.* 380,000 metagenomic sequence reads using MG-RAST [79] indicated the presence of ssRNA virus sequences. Contigs were assembled using the meta-assembler workflow in CAMERA [80].

Repeated terminal sequences in the contig indicated a circular genome. To confirm circularity and to rule out artificial chimera formation either by ϕ29 polymerase amplification [81,82] or spurious assembly, inverse PCR was used to amplify the complete virus genome from the BSL DNA sample that had neither been amplified with ϕ29 polymerase nor used for pyrosequencing. The reverse and forward primers (5’-CCTATTGGTGAGCTGTGGGTTGA-3’ and 5’-GTATCGCGTAACTTTAAGGAAACCG-3’) were used to amplify the complete circular genome. Extension from inverse PCR primers originates within the capsid protein ORF. The 4089 nucleotide virus genome was amplified with Phire polymerase (Finnzymes) [98°C, 30 sec. initial denaturation, followed by a touch-down stage of 8 cycles; 98°C, 5 sec. denaturation, 72°C to 65°C, 5 sec. annealing decreasing 1°C/cycle, 72°C extension for 1.5 min. followed by 25 cycles of amplification; 98°C, 5 sec. denaturation, 65°C, 5 sec. annealing, 72°C, 1.5 min. extension, followed by a final 72°C extension for 3 min.] The whole-genome PCR product was cloned into pCR-TOPO-Blunt (Invitrogen) using the manufacturer’s instructions. Plasmid DNA was used for Sanger sequencing. The BSL RDHV genome sequence has been submitted to GenBank: accession number JN900499.

The highly conserved DNA stem loop (Figure 1B) in the BSL RDHV genome was detected using the Mfold v4.6 nucleic acid folding and hybridization web server by applying default settings at 70°C [83]. ORFs were predicted using Mold, Protozoan and Coelentrate codon tables, and related virus sequences were retrieved by two PSI-BLAST search iterations of NCBI nr/nt and env databases using default parameters (threshold = 0.005). Unrelated *Microviridae* Rep sequences were manually chosen for comparison. The CP and Rep ORFs were compared against selected sequences using BLASTp to prepare BLASTp tables (Figures 2 and 5).

The 542 amino acid BSL RDHV capsid protein ORF sequence was aligned to closely related sequences retrieved from PSI-BLAST searches using ClustalW with default parameters in MEGA v.5 [84] (Pairwise alignment: gap opening penalty = 10, gap extension penalty = 0.1. Multiple alignment: gap opening penalty = 10, gap extension penalty = 0.2. Protein weight matrix = Gonnet. Delay divergent cutoff = 30%) and then refined by hand. The phylogenetic tree was inferred using the Neighbor-Joining method [85] by applying a bootstrap test with 1000 replicates [86]. The 418 amino acid replicase ORF sequence alignment and phylogenetic tree was prepared using the same parameters as in the CP alignment. Both CP and Rep ClustalW multiple sequence alignment figures were prepared using Jalview [87].

Each ORF was analyzed using InterProScan [88,89] to locate conserved protein domains within each ORF. Tertiary protein structures were first predicted by threading using the CPH Model Server [90], which automatically selects an appropriate solved protein structure as a scaffold. Structure predictions were confirmed and refined using EsyPred3D [91], by entering Protein Data Bank (PDB) structure scaffolds manually. Structure predictions of BSL virus proteins were compared to solved crystallographic structures using the MultiSeq application [92] in VMD [93] (Figures 7A and 7B).

## Abbreviations

BSL = Boiling Springs Lake; CP = Capsid Protein; ds = double-stranded; GOS = Global Ocean Survey; ORF = Open Reading Frame; PDB = Protein Data Bank; RCRE = Rolling circle replicase endonuclease; RdRp = RNA-dependent RNA polymerase; Rep = Replicase; RT = Reverse Transcription/Transcriptase; ss = single-stranded; S3H = Superfamily-3 helicase; TFF = Tangential Flow Filtration.

## Competing interests

The authors declare that they have no competing interests.

## Authors’ contributions

GSD drafted the manuscript, initiated investigation of the BSL RDHV data, prepared the BSL metagenomic sample, performed experiments and analyses, and prepared figures. KMS performed separate corroborative analyses, initiated and oversaw the project, obtained all necessary grants, permits and resources, and edited the manuscript. All authors read and approved the final draft of the manuscript prior to submission.

## Authors’ information

Reviewers’ reports

**Reviewer's report 1:Dr Eugene Koonin (National Center for Biotechnology Information, USA)**

This is a truly exciting paper that reports the discovery of a completely unexpected entity, an apparent hybrid between a ssDNA virus related to circoviruses and an RNA virus related to tombusviruses. This finding is of great interest on two levels. First, to my knowledge, such a chimera between RNA and DNA viruses – not only of these particular families but in general - has never been observed before. Of course, there are many examples of mixing and matching in the virus world, but somehow they so far have been confined to the same type of nucleic acid. Second, this work highlights the new route to discovery in virology – the metagenomic path. This is literally a fishing expedition, with all its advantages and drawbacks. The main advantage is the capacity to discover essentially everything that is ‘out there’, even at low abundance, without the need for the laborious and biased procedures of virus and host growth. But, here is also the severe limitation of metagenomics: neither the host nor, strictly speaking, the virus is identified to the regular standards of microbiology and virology. In any case like this, but most especially when a bizarre chimera was discovered, it is crucial to show as convincingly as possible that the presented sequence is indeed the virus genome rather than some assembly artefact or chimeric clone. I think this is done in a satisfactory manner in this paper, by inverse PCR from an independent environmental sample. So I believe this is a real virus. Moreover, it is remarkable that the closest homologues of both the Rep protein and the capsid protein were detected in other metagenomic samples, those from the GOS. It is extremely intriguing whether these represent the same kind of chimeric genomes or the proposed RNA-DNA recombination event is relatively recent, and these neighbors are the closest relatives from the respective families of RNA and DNA viruses. With the genome of BSL-RDHV released, this should not be too hard to test. In a more general plane, one cannot help wondering how many of such unexpected wonders of the virus world await in all kinds of environments, and more practically, are the criteria for recognizing a new virus are going to change any time soon.

I have some minor specific issues with the paper.-The title may be construed as a bit misleading as ‘evolutionary link’ seems to imply that ssDNA virus(es) evolved from ssRNA virus(es) or vice versa. I would suggest mentioning the chimeric genome in the title itself.

***Author’s response****: The title has been revised.*

-I am surprised by the methodology employed for building the trees (‘rough-cluster cladograms’) in Figure 3. Why use this crude approach instead of regular maximum likelihood method (RaxML) and perhaps even a Bayesian method in addition? Not that I expect the result to change dramatically but the new virus is interesting and unusual enough to invest a reasonable effort to make the phylogenetic analysis as robust as possible.

***Author’s response****: This section has been revised and much more extensive alignments are presented and phylogenetic analysis performed (Figures*3*,*4*and*6*).*

-I find the emphasis on the similarity in genome organization between the circular ssDNA virus which BSL-RDHV apparently is and ssRNA tombusviruses to be rather strange. Isn’t the similarity with circoviruses much more straightforward? To me, this looks like a circovirus in which the capsid protein was displaced by one from a tombus-like virus.

***Author’s response****: This has been revised throughout the text. However, we find the genome arrangement to be strikingly different from most circoviruses, thus have retained Figure*1.

**Reviewer's report 2:Dr. Mart Krupovic (nominated by Dr. Patrick Forterre) (Institut Pasteur, France):**

Diemer and Stedman report on characterization of a putative viral genome, which has been obtained in the course of a metagenomic analysis of virome samples collected at the Boiling Springs Lake. The putative viral genome (BSL-RDHV) encodes four proteins, two of which share sequence similarity with proteins from previously characterized viruses. One of these proteins is related to typical superfamily II rolling circle replication initiation proteins that are abundantly found in DNA viruses and plasmids. Strikingly, the other one is most similar to capsid proteins of eukaryotic icosahedral positive-sense RNA viruses. The observation that genes for two key viral functions— virion formation and genome replication—are apparently derived from unrelated RNA and DNA viruses/replicons to form a new chimeric viral entity is exciting, although not entirely novel (see below). The findings presented in this paper substantially advance our understanding not only on the genetic diversity in the virosphere but also on the potential mechanisms responsible for the emergence of novel viral types. I therefore think that the paper is definitely worth publishing. However, some parts of the manuscript can still be improved as detailed below.

Background: This section consists of five lines praising the usefulness of metagenomics in studying virus evolution, followed by a few paragraphs, which resemble Results rather than the Introduction. Given the fact that the paper is about virus evolution, the Background section could provide some information on the current hypotheses on the origin of viruses and the mechanisms of their evolution. This would allow the readers to more fully appreciate the significance of the findings presented in the Results section The authors might find useful the recent reviews on this subject by (Koonin and Dolja, 2011; Krupovic et al., 2011; Forterre and Prangishvili, 2009). Dolja VV, Koonin EV: Common origins and host-dependent diversity of plant and animal viromes. Curr Opin Virol 2011, 1(5):322–31. Krupovic M, Prangishvili D, Hendrix RW, Bamford DH: Genomics of bacterial and archaeal viruses: dynamics within the prokaryotic virosphere. Microbiol Mol Biol Rev 2011, 75(4):610–35. Forterre P, Prangishvili D: The origin of viruses. Res Microbiol 2009, 160(7):466–72.

***Author’s response****: This section has been extensively revised.*

Results: I. Capsid protein: Similarity of the BSL-RDHV capsid protein to those of RNA viruses appears to be highly significant (especially to the CP of Sclerophthora macrospora virus A). However, the similarity is confined to domains S and P of the RNA virus CPs, which covers only the central region of the BSL-RDHV capsid protein (residues 156–302). The BSL-RDHV is 542 aa. Could the authors comment on the N- and C-terminal regions of the BSL-RDHV CP, which are not shown in the alignment presented in Figure S1?

***Author’s response****: We have revised the text to discuss these aspects and have included tables of BLASTp hits and extensive alignments (Figures*2*–*6*).*

Do these regions share sequence similarity to proteins in the databases? What is their predicted secondary structure? Are they likely to fold into independent functional domains? How this might affect capsid formation? In addition, the authors should provide more information on Sclerophthora macrospora virus A (SmV-A) and Plasmopara halstedii virus A (PhV-A), the two viruses sharing the highest sequence similarity with the CP of BSL-RDHV. Stating the fact that they are unclassified ssRNA viruses is not enough. For example, what is the host range of SmV-A and PhV-A (if known), what is the genomic relationship between these viruses and tembusviruses, etc.

***Author’s response****: This section has also been revised and we hope that this work will stimulate research on the under-studied SmV-A and PhV-A viruses, since they may also provide insight into the mechanism of formation of the BSL RDHV-like virus genomes.*

Perhaps this information might provide some hints about the origin of BSL-RDHV? The S-P domain organization is not typical for all icosahedral (+)ssRNA viruses. The information on how widespread this CP architecture is among RNA viruses would be very interesting. Is it only found in Tombusviridae and a few unclassified viruses?

***Author’s response****: This S-P configuration is only known and demonstrated by X-ray crystallography in the “carmovirus-like” group of Tombusviridae.*

From the alignment (Figure S1) it seems that the S domain is considerably more conserved between BSL-RDHV and tombusviruses. Does the same hold true when BSL-RDHV CP is compared with SmV-A and PhV-A only?

***Author’s response****: As above, this section has been considerably revised.*

Besides, the S-P organization is not called “double jelly-roll configuration”, as the authors state on page 5. Double jelly roll fold is found in diverse dsDNA viruses and is structurally quite different from that of the CP of tombusviruses (Krupovic and Bamford, 2008). Krupovic M, Bamford DH: Virus evolution: how far does the double beta-barrel viral lineage extend? Nat Rev Microbiol 2008, 6(12):941–8.

***Author’s response****: This has been corrected*.

In addition, the Qres colouring of the CP model in Figure 2A is not very meaningful and can be eliminated.

***Author’s response****: We find that, since the alignment does not indicate a high degree of amino acid sequence similarity in the P domain of the CP proteins, a structural assessment is warranted to better substantiate claims of interviral transfer and homology of the BSL and S-P-type CPs of tombusviruses. That the structural conguency extends over the whole structure is best displayed with a Qres score.*

II. Rep protein: The authors could briefly introduce the rolling circle replication initiation proteins (RCR Reps). RCR Reps contain three conserved motifs (not just active site Tyr): Ilyina TV, Koonin EV: Conserved sequence motifs in the initiator proteins for rolling circle DNA replication encoded by diverse replicons from eubacteria, eucaryotes and archaebacteria. Nucleic Acids Res 1992, 20(13):3279–85. Are all three motifs conserved in the BSL-RDHV? Figure S2 shows an alignment between the nuclease domains of RCR Reps from BSL-RDHV and PCV2 (by the way, the legend does not correspond to this figure). A more inclusive set of RCR Reps could be compared (and not only for the nuclease, but for the helicase domain as well).

***Author’s response****: See revised Figure*6.

In addition, the fact that there is a stem loop preceding the Rep gene does not necessarily suggest the single-stranded nature of the BSL-RDHV genome in the virion (page 6, second paragraph). dsDNA viruses also use RCR Reps for replication (e.g., corticovirus PM2).

***Author’s response****: While it does not completely rule out the possibility that the BSL RDHV virus harbors a double-stranded genome within the virion, the stem-loop, the sequence similarity to the PCV Rep, and the Rep structural assessment all strongly indicate a single-stranded circovirus-like genome and replication cycle. Until virions can be produced and DNA extracted for analysis, this cannot be definitively shown. Experiments to detect ssDNA in BSL samples are underway. Moreover, no detectible sequence similarity between the BSL/circoviral and PM2 Rep was detected, and no nucleic acid sequence similarity was detected between the BSL and PM2 origins of replication, indicating that the BSL virus is not likely to be related to the PM2 corticovirus.*

III. The trees: I suggest replacing the rough-clustering trees (Figure 3) with corresponding alignments, since such trees are not very meaningful. Figure 3A shows the CP tree of BSL-RDHV, tombusviruses, satellite viruses, geminiviruses and nanoviruses. The authors say that BSL-RDHV clusters with tombusviruses, “to the exclusion of capsid proteins found in ssDNA plant-infecting viruses that also encode Rep”. None of these other proteins (for which information on the structure is available) possess both S and P domains, while the information on the nanovirus CP, to my knowledge, is not available at all. It therefore makes no sense to put on the same tree proteins that might not even be homologous. Similarly for Figure 3B, which shows the tree of RCR Reps – the similarity between the Reps of microviruses and circoviruses is confined to the three motifs of the nuclease domain (microviral Rep also does not have the helicase domain). Supplementary files 2 (Blast scores) and 3 (accession numbers) should be combined. It would be also useful if the authors could supplement the table with the pairwise identity values.

***Author’s response****: This has been done.*

Conclusions: “…RNA-DNA recombination has only been inferred”: Perhaps it could be mention here that numerous RNA virus genomes (from different families) were recently discovered in the genomes of various eukaryotic hosts, which suggests that RNA-DNA recombination might be not as uncommon as previously believed.

***Author’s response****: This has been added and see the author’s response to Reviewer 3.*

The authors point out “that lateral transfer of capsid genes occurred between an ancestor of ssRNA satellite viruses and a circular, ssDNA geminivirus or nanovirus during co-infection [32]”. However, what we suggested in ref [32] is that geminiviruses originated from plasmids of phytopathogenic bacteria (phytoplasma) by acquiring the capsid-coding gene from a plant-infecting RNA virus, i.e., recombination occurred between two unrelated DNA (plasmid) and RNA (virus) replicons to give rise to a novel element – the ancestor of geminiviruses. The claim that “ssRNA satellite virus capsid proteins are found exclusively in ssDNA genomes of the large and well characterized Geminiviridae and Nanoviridae families” is also not supported: (i) there is no evidence that nanoviral CP adopts the jelly-roll fold (even though this is probably true), (ii) among DNA viruses this fold is not restricted to geminiviruses as it is also found in CPs of parvoviruses and microviruses (and certain dsDNA viruses), (iii) most importantly, the single jelly roll fold is most widespread in viruses with RNA genomes (12 different families!). The suggestion that “ssRNA satellite viruses most likely acquired their capsid proteins from gemini- and nanoviruses” has no ground. The fact that “Satellite, gemini- and nanoviruses often co-infect the same hosts” per se is not a proof, especially considering that the primary partner during coinfection for ssRNA satellite viruses are other ssRNA viruses (with jelly roll CPs).

***Author’s response****: We have elected to remove this particular example as a possible precedent for interviral RNA-DNA recombination because the claims asserted in Krupovic et.al.,2009 have not yet been substantiated. We agree that the jelly-roll fold itself probably originated in RNA viruses, and that a CP gene phylogeny indicates a common ancestry amongst the RNA satellite-, DNA gemini- and nanovirus CPs. However, we find the assertion that the gemini- and nanovirus CPs were directly and recently obtained from an RNA satellite-like virus to be speculative. While investigating the evolutionary trajectories of the jelly-roll fold and determining its ultimate origin in DNA virus groups is certainly an intriguing prospect, such an endeavour is beyond the scope of this report.*

The authors prefer a scenario according to which “the capsid gene was transferred from a ssRNA virus to a ssDNA virus in the predecessor of the putative RDHV family”. However, can the authors be sure that at the origin of the RDHV ancestor was a virus and not a plasmid? In principle, the acceptor of the tombusvirus-like capsid gene could have been any kind of a replicon (e.g., a plasmid) with a circovirus-like RCR Rep. Besides, plasmids could have also been at the origin of circoviruses, as we have pointed out previously.

***Author’s response****: The BSL Rep protein sequence bears little resemblance to plasmid Reps, while demonstrating a substantial similarity to circovirus-like Reps. Unless there are other uncharacterized plasmids with circovirus-like Reps, the data indicate that it is more likely that the recombination occurred in a circovirus-like genome. While it is conceivable that circoviruses ultimately originated from plasmids, the low level of sequence divergence between the BSL RDHV Rep, CP and other related proteins indicate a recent acquisition of the CP protein by an already circovirus-like ancestor. The alternative hypothesis would require the convergent evolution of the BSL and tombusvirus-like CPs, which we consider highly unlikely.*

Last paragraph of the Conclusions: In my opinion, it is an overstatement to say that the observations presented in this paper implicate viruses in the transition from the RNA-World to the DNA World.

***Author’s response****: This section of the conclusion has been modified for clarity, but we would like to confirm our difference of opinion on this subject.*

However, I certainly agree that the findings “extend the modular theory of virus evolution to encompass a much broader range of possibilities”. What I also find intriguing about such chimeric viruses is how their discovery might impact our views on the timeline of virus origins as well as our attempts to devise higher levels of virus classification. It is often assumed that viruses emerged around the same time or even before the cellular organisms while the possibility that new groups of viruses might be emerging in the contemporary biosphere is rarely discussed. Building on the hypothesis by Koonin and Ilyina (1992), we have suggested that geminiviruses might represent one such group of “new” viruses [32]. Koonin EV, Ilyina TV: Geminivirus replication proteins are related to prokaryotic plasmid rolling circle DNA replication initiator proteins. J Gen Virol 1992, 73:2763–6. RDHV might be an even more convincing example in support of the on-going emergence of novel virus groups from pre-existing mobile genetic elements (viruses and plasmids).

***Author’s response****: We very much agree with your assessment.*

For higher-order virus classification, I personally favour the capsido-centric view (Krupovic and Bamford, 2009, 2010), according to which determinants for virion architecture are inherited in a given viral group from their common ancestor, while genetic determinant for other functional modules (e.g., for genome replication proteins) move relatively freely in and out of these viral genomes. In other words, the movement of functional modules occurs relative to the capsid-encoding genes. Krupovic M, Bamford DH: Does the evolution of viral polymerases reflect the origin and evolution of viruses? Nat Rev Microbiol 2009, 7(3):250. Krupovic M, Bamford DH: Order to the viral universe. J Virol 2010, 84(24):12476–9. In contrast, according to another line of thought, different functional modules in the viral genomes deserve equal weight when considering relationships between viruses: Koonin EV, Wolf YI, Nagasaki K, Dolja VV: The complexity of the virus world. Nat Rev Microbiol 2009, 7(3):250. Lawrence JG, Hatfull GF, Hendrix RW: Imbroglios of viral taxonomy: genetic exchange and failings of phenetic approaches. J Bacteriol 2002, 184(17):4891–905. Therefore, depending on the viewpoint, RDHV can be considered as a relative of tombusviruses, which had its original genome replication machinery (RdRp) replaced with a gene for RCR Rep. On the other hand, it might also be seen as a circovirus in which the ancestral CP gene was replaced by a gene from a tombusvirus. What do the authors think about classification (and affiliation to the existing viral taxa) of RDHV and other chimeric viruses, which are likely to be discovered in the future?

***Author’s response****: These points are highly intriguing to consider and this commentary is very much appreciated. First, the continued use of metagenomics promises to have a marked effect on current schemes of virus taxonomy. We may only guess at what effects the BSL RDHV virus and its relatives will have on these taxonomic frameworks. Second, this issue pertaining to the trajectories of the Rep and CP modules brings to the fore an important question regarding the origin of the linear and circular ssDNA viruses. It is unlikely that the BSL RDHV-like genome evolved incrementally from an RdRp-containing RNA virus. However, the notion that linear and circular ssDNA viruses first evolved from ssRNA viruses in such a manner, first by conversion to DNA and then via the acquisition of an RCRE domain (the Rep S3H domain also being derived from an RNA virus), as opposed to having emerged largely via modular exchanges is certainly a topic very worthy of investigation.*

**Reviewer's report 3:Dr. Arcady Mushegian (University of Kansas School of Medicine, USA)**

The manuscript by Diemer and Stedman reports the existence of the new virus, characterized by circular single-strand DNA genome and a novel configuration of two genes, i.e., 1. nanovirus- or circovirus-like replication protein with the usual predicted DNA-nicking and NTPase domains and 2. the jelly-roll capsid protein clearly related to capsid proteins of positive-strand RNA viruses (tombusvirudae) and two unclassified RNA viruses of fungi. The experiments indicate that the metagenomic sample from the hot lake contains the full circular genome, and that similar genomes most likely exist in the ocean samples (in that case, their circular form was not shown, but most likely will be). This is a fascinating discovery of a novel virus group, suggesting the ancient act of exchange of genetic material between RNA and DNA virus genomes. I fully support the publication of this study, but must request that some of the more sweeping statements in the paper are moderated, in order to better agree with the evidence. Abstract: “little is known about their collective origin and evolutionary history” --- see next comment. Ibid. “it is not currently possible to determine whether the principal virus groups arose independently, or whether they have a shared evolutionary history” --- the hypothesis that RNA viruses arose before the advent of DNA genomes, when the protein-encoding genomes were made of RNA, is not unreasonable. This would argue for the independent, or at least separate in time, origins of DNA and RNA virus genomes. Therefore, the word 'collective' in the first sentence is doing some heavy lifting that it probably should not. On the other hand, retro-transcribing viruses and RNA viruses seem to satisfy anyone's definition of two 'principal virus groups', and yet there is plenty of evidence that they have a shared evolutionary history, at least in their replication enzyme.

***Author’s response****: This section has been extensively revised.*

Ibid. “no mechanism for RNA-DNA recombination has yet been identified” --- what about retrohoming of group II introns ?

***Author’s response****: The following passage was added to the conclusions section based on the suggestions made by Mushegian and Krupovic: ”The presence of non-retroviral RNA virus genes in cellular genomes [*[61-66]*] suggests that some cellular mechanism exists that allows RNA-DNA recombination in lieu of a virus-derived RT. Although the group II intron retro-homing phenomenon [*[67]*] and transposon mediated exchanges have not been observed to mediate interviral lateral gene transfer, these or similar host cell-based mechanisms may have facilitated the formation of the BSL RDHV-like viruses.”*

p. 5: The moniker “RNA-DNA hybrid virus” (RDHV) must go. This is a thoroughly misleading name. The authors show abundant evidence of a circovirus-like or nanovirus-like virus with single-strand DNA genome that, in the past, have acquired a capsid protein from an RNA virus. Nonetheless, it is a DNA virus now. This is not even the first example of that kind of mosaicism – BL1/BC1 proteins of bipartite geminiviruses are similar to the 30 K family of movement proteins of plant RNA viruses, but no one calls bipartite geminiviruses “DNA-RNA viruses” because of that. RNA genomes of closteroviruses encode homologs of cellular HSP70 proteins, but these viruses are not RNA-DNA viruses either. Descriptive name such as “Boiling Spring Lake Virus 1” or something of this kind should do just fine. Note that this objection to “RDHV” is not the nomenclature war, but rather aims at setting the molecular record straight.

***Author’s response****: The moniker “RDHV” is mentioned in the text as provisional. We feel that a succinct descriptive name for this new virus genome-type is warranted, at least temporarily. Other conceivable names seem insufficient to describe a novel and probably wide-spread virus group and its ancestry, and would be significantly more confusing or excessively complicated (e.g. “a Boiling Springs Lake virus from the Sargasso Sea”). We completely agree that the genome discovered represents a DNA virus. Once we have identified the host and/or structure for the virus we will propose a taxonomically appropriate name through the ICTV (and let the nomenclature wars rage).*

p. 5 and later: I am sure that there is a straightforward sequence-similarity argument on the evolutionary relatedness of “RDHV” capsid protein and tombusviruses. I could obtain statistically significant similarity between the former and the latter by PSI-BLAST and HHPred approaches. I recommend that the authors do the same. Instead, we are reading “The predicted structure of the BSL RDHV capsid protein is congruent to the S-P domain double jelly-roll configuration found in the ssRNA Tomato Bushy Stunt (TBSV) and Melon Necrotic Spot (MNSV) tombusviruses [12,13]. Amino acid sequences are moderately conserved amongst the three proteins based on BLOSUM80 [14], while percent sequence identity is low (Figure 2A) (see Additional Figure 1 for alignment).” This is ambiguous: if the sequence-similarity/database search statistics arguments (not the same as sequence identity!) are not sufficient to establish the evolutionarily significant similarity, then there is no basis for threading and structure modeling; and if sequence-similarity arguments were used, why not say so?

***Author’s response****: This section has been extensively revised and Figures added (Figures*2*–*6*).*

p. 7: “The most parsimonious scenario” --- more parsimonious than which other scenarios?

***Author’s response****: This section has also been revised. See reply to Krupovic in regard to the origin of linear and circular ssDNA viruses.*

pp. 7–8: Several mentions of satellite RNA viruses seem out of place – tombusviruses are not satellites and neither are fungal viruses discussed in the paper?

***Author’s response****: These references have been clarified.*

pp. 8–9: (last paragraph of the paper) “Assuming that RNA viruses evolutionarily preceded all DNA virus groups[33,34], evidence of gene transfer from RNA to DNA viruses complements the RNA-first theory[35].” --- I do not understand what this means. First, if we assume that RNA viruses evolutionarily preceded all DNA virus groups, then we do have a partial answer to the question that was said to be currently impossible to answer in the Abstract (see above). Second, “to complement” more or less means to provide a missing part or an additional, compatible line of argument, correct? I am not sure what does the virus described in this study have to do with evolutionary precedence of RNA viruses over DNA viruses: surely, in order for this virus to emerge, both RNA viruses and DNA viruses have to be around already?

***Author’s response****: This final paragraph has been revised and clarified.*
